# Graphene plasmonically induced analogue of tunable electromagnetically induced transparency without structurally or spatially asymmetry

**DOI:** 10.1038/s41598-019-56745-9

**Published:** 2019-12-30

**Authors:** Yuwen He, Jianfa Zhang, Wei Xu, Chucai Guo, Ken Liu, Xiaodong Yuan, Zhihong Zhu

**Affiliations:** College of Advanced Interdisciplinary Studies, National University of Defense Technology, Changsha, 410073 People’s Republic of China

**Keywords:** Nanophotonics and plasmonics, Two-dimensional materials, Optical properties and devices

## Abstract

Electromagnetically induced transparency (EIT) arises from the coherent coupling and interference between a superradiant (bright) mode in one resonator and a subradiant (dark) mode in an adjacent resonator. Generally, the two adjacent resonators are structurally or spatially asymmetric. Here, by numerical simulation, we demonstrate that tunable EIT can be induced by graphene ribbon pairs without structurally or spatially asymmetry. The mechanism originates from the fact that the resonate frequencies of the bright mode and the dark mode supported by the symmetrical graphene ribbon pairs can be respectively tuned by electrical doping levels, and when they are tuned to be equal the graphene plasmon coupling and interference occurs. The EIT in symmetrical nanostructure which avoids deliberately breaking the element symmetry in shape as well as in size facilitates the design and fabrication of the structure. In addition, the work regarding to EIT in the structurally symmetric could provide a fresh contribution to a more comprehensive physical understanding of Fano resonance.

## Introduction

Electromagnetically induced transparency (EIT) is a concept originally observed in atomic physics where the coherent coupling of a broad and a narrow resonance leads to quantum interference^1,2^. This concept was later extended to classical optical systems and induced by interference between a bright mode in one optical resonator and a dark mode in an adjacent optical resonator^3,4^. With the characteristic of high quality factor, steep dispersion and near field-enhanced, EIT has many applications ranging from signal processing^5^, sensors^6–8^, lasing^9,10^, nonlinear^2,11^ and slow-light devices^12,13^. Recently, metal nano-plasmonic structures have attracted great attention due to their ability of confining light to sub-wavelength dimensions and opening up the possibilities to construct optical devices with various scales and shapes while maintaining consistent optical properties that do not depend on the dimensions of the device. Many metallic plasmonic structures have been designed to achieve the EIT^8,14–20^. A direct effective way for inducing EIT is destroying the symmetry in metallic structures. For example, by deliberately breaking the element symmetry in shape as well as in size, the concentric double rings^21–23^, ring-disk composite^24^, asymmetric split-ring pairs^25^, and mismatched nanoparticle pairs^26^ can induce EIT. Although most of these symmetry breaking nanostructures exhibit obvious EIT, they are complex in structure, difficult to design and sensitive to preparation accuracy. The EIT with simple structure and high preparation tolerance such as general symmetrical structures are desirable. However, owing to the difficulty in generation of spectrally overlapping plasmonic resonances with very different radiance in highly symmetric nanostructures, there’re few reports about the EIT based on highly symmetric plasmonic nanostructures. In addition, metallic plasmonic nanostructures are hard to tune due to the limitation of material, which make it usually necessary to refabricate the physic structure in order to achieve the tunability. But, in many practical applications, it’s hard to change the physical structure after manufacturing. So, it is meaningful and challenging to get tunable EIT without structurally or spatially asymmetry.

Fortunately, graphene^27^, a novel semi-metal material, has attracted tremendous attention due to its unique optical and electrical properties, such as low loss, ability to support plasmonic resonance in the terahertz and mid-far infrared bands^28–30^, strong field localization^31^ and nonlinear optical effects^32^. So far, graphene has widely applications on photonics devices ranging from ultrafast pulsed lasers^33–37^, graphene polarizer^38,39^ and light emitting devices^40^. More importantly, with electrical gating^41,42^ and chemical doping^43^, graphene shows the ability to tune plasmonic resonance. Some researchers have proposed and studied various graphene structures to provide tunable plasmonic resonance, for instance, graphene film^44^, graphene nanorings^45,46^, graphene patches^47^, graphene photonic crystal structures^48^. Specially, nanorings and graphene patches have been used to design the tunable asymmetric EIT structure^45–47^.

In this work, we demonstrate that tunable EIT can be induced by graphene ribbon pairs without structurally or spatially asymmetry. Here, the reason for using graphene ribbon pairs is that the graphene ribbon is the simplest form of sub-wavelength graphene plasmon structure and has been widely fabricated by different researchers^28^, and can easily realize different Fermi energy on different ribbons at the same time. We elucidate the physical mechanism firstly. Then, we present numerical simulations and results. Finally, we investigate the effects of some important structural parameters. It is worth mentioning that our structure can not only utilize graphene as the plasmonic platform to excite EIT, but also use other two dimensional materials like black phosphorus (BP)^49–51^ and antimonene^52^.

## Structure and Principle

**Figure 1:**
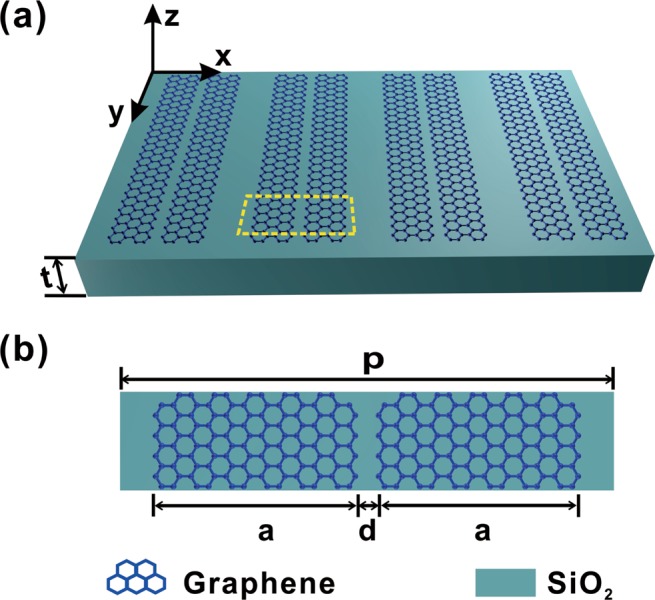
(**a**) The schematic diagram of arrays of graphene ribbon pairs without structurally or spatially asymmetry supported by a piece of dielectric substrate with semi-infinite thickness $t$. (**b**) Top view of a unit cell. The periodic interval, ribbon width, and ribbon edge to edge distance are $p$, $a$, $d$, respectively.

The proposed structure is presented in Fig. 1, which consists of arrays of graphene ribbon pairs supported by a piece of dielectric substrate. The structure is characterized by the periodic interval $p$ of graphene ribbon pairs with ribbon width $a$, ribbon edge to edge distance $d$. The two ribbons of graphene ribbon pairs are respectively electrical doped, the corresponding Fermi energy being ${E}_{F1}$ and ${E}_{F2}$. The thickness *t* of the dielectric substrate is set to be semi-infinite.

The doped graphene behaves as an essentially 2D electronic system. So, the electromagnetic waves coupled to collective charge excitation (plasmons) can be excited in the doped graphene. The plasmons become localized in the patterned graphene because of the confinement of spatial dimension. The localized plasmons correspond to collective charge oscillation modes of various orders of standing waves. Therefore, the doped graphene ribbons providing confinement along one spatial dimension can be predicted to produce sharp plasmon resonances across the width of ribbons^53,54^. The corresponding N-order graphene plasmon resonance of a separate ribbon occurs at

1 $$a=(N-\phi /\pi ){\lambda }_{eff}/2,$$

where *N* is a positive integer determining the order of a resonance mode, $\phi$ is the phase of the reflection coefficient for plasmon reflection at ribbon terminations and ${\lambda }_{eff}$ is the effective resonance wavelength. ${\lambda }_{eff}$ is determined by the real part of permittivity of graphene plasmon *Re*$({n}_{eff})$ and is rewritten as

2 $${\lambda }_{eff}={\lambda }_{0}/{Re}({n}_{eff}),$$

where ${\lambda }_{0}$ is the vacuum wavelength. In the considered frequency range, the intraband response dominates the conductance, so^55^

3 $${Re}({n}_{eff})\approx \hslash \omega ({\varepsilon }_{1}+{\varepsilon }_{2})/(4{\alpha }_{0}{E}_{F}),$$

where *ħ* is the reduced Planck’s constant, for the graphene ribbons with one side exposed to the surrounding medium with dielectric constant ${\varepsilon }_{1}$ and another side exposed to the substrate with dielectric constant ${\varepsilon }_{2}$. ${\alpha }_{0}$ is the fine-structure constant and ${E}_{F}$ is the Fermi energy of graphene ribbon. From Eqs. (1)–(3), by simple algebra operation, the resonant frequency for the N-th order plasmon resonance mode can be obtained as

4 $$f\approx \sqrt{c{\alpha }_{0}{E}_{F}(N-\phi /\pi )/\pi \hslash a({\varepsilon }_{1}+{\varepsilon }_{2})},$$

where *c* is the light speed of the vacuum.

From Eq. (4), we find the graphene plasmon resonance frequency is determined by the Fermi energy ${E}_{F}$ and the width $a$ of the graphene ribbons. In our model system, the two ribbons of a graphene ribbon pair have the same width $a$ but electrically doped by different Fermi energy ${E}_{F1}$ and ${E}_{F2}$. Therefore, the graphene ribbon pairs can support two different order plasmon resonance modes operating at the same frequency ${f}_{0}$ at the same time, which satisfies

5 $$\begin{array}{rcl}{f}_{0} & \approx  & \sqrt{c{\alpha }_{0}{E}_{F1}({N}_{1}-{\phi }_{1}/\pi )/\pi \hslash a({\varepsilon }_{1}+{\varepsilon }_{2})}\\  & = & \sqrt{c{\alpha }_{0}{E}_{F2}({N}_{2}-{\phi }_{2}/\pi )/\pi \hslash a({\varepsilon }_{1}+{\varepsilon }_{2})},\end{array}$$

where ${N}_{1}$ and ${N}_{2}$ are the order number of two different resonance mode, and ${\phi }_{1}$ and ${\phi }_{2}$ are the phase of the reflection coefficient for two different plasmon reflection at ribbon terminations, respectively. When the ribbon edge to edge distance $d$ is appropriately small, near field coupling and interference can occur between the two different order plasmon resonance modes. If one of the two resonance modes is bright mode and the other is dark mode, EIT occurs. This means that EIT can be induced by graphene ribbon pairs without structurally or spatially asymmetry.

## Results and Analysis

To verify the theoretical prediction, we next conduct full-wave numerical simulations employing frequency domain solver in CST Microwave Studio. In our simulation, the dielectric substrate is set to be a normal non-dispersive material with relative permittivity ${\varepsilon }_{2}=2.25$ and relative magnetic permeability ${\mu }_{r}=1$. In terahertz and mid-far infrared bands the in-band transition of graphene dominates, and the surface conductivity of graphene follows the Drude-like expression^56^

6 $${\sigma }_{g}(\omega ,{E}_{F})=\frac{{e}^{2}{E}_{F}}{\pi {\hslash }^{2}}\frac{i}{\omega +i{\tau }^{-1}},$$

where ${E}_{F}$ respects the Fermi energy of graphene relating to carrier concentration $n$ (${E}_{F}=\hslash {v}_{F}\sqrt{\pi n}$). $\omega$ is the frequency and $\tau =\mu {E}_{F}/e{v}_{F}^{2}$ is the carrier relaxation time, in which $\mu =10000\,c{m}^{2}/(V\cdot s)$ is the carrier mobility, ${v}_{F}={10}^{6}\,m/s$ is the Fermi velocity. The thickness of the single-layered graphene ribbons is set as ${t}_{g}=1\,nm$, and the equivalent permittivity of graphene can be derived from $\varepsilon ^{\prime} =\varepsilon +i{\sigma }_{g}/\omega {t}_{g}$^57^.

**Figure 2:**
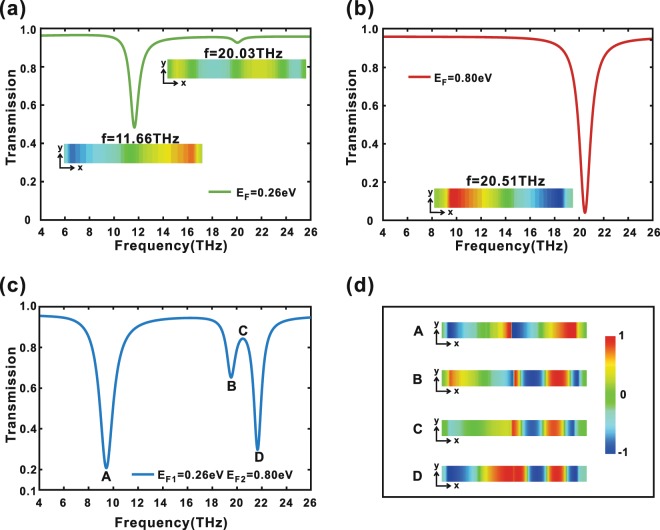
The transmission spectrum of the case of arrays of single graphene ribbon. The Fermi energy of graphene ribbon is set to (**a**) ${E}_{F}=0.26\,eV$ and (**b**) ${E}_{F}=0.80\,eV$, respectively. Insets: electric field distribution at the resonance frequency. The distributions of the $\hat{{\rm{z}}}$ component of electric field on the cutting plane perpendicular to $\hat{{\rm{z}}}$ direction at a distance of $10\,nm$ beneath graphene sheet at corresponding transmission dips. (**c**) The transmission spectrum of the case of arrays of symmetric graphene ribbon pairs with two ribbons doped by different Fermi energy $0.26\,eV$ and $0.80\,eV$. (**d**) The distributions of the $\hat{{\rm{z}}}$ component of electric field on the cutting plane perpendicular to $\hat{{\rm{z}}}$ direction at a distance of $10\,nm$ beneath graphene sheet corresponding to transmission dips A, B, D, and peak C, respectively.

First, we consider the case of arrays of single graphene ribbon where $p=1360\,nm$, $a=580\,nm$, ${\varepsilon }_{1}=1$ and ${E}_{F}=0.26\,eV$ or ${E}_{F}=0.80\,eV$. Figure 2 shows the calculated transmission spectra under normal incident wave with the electric field parallel to $\hat{{\rm{x}}}$-axis. From Fig. 2, we can see there are two deep transmission dips at $11.66\,THz$ and $20.51\,THz$ that correspond to the Fermi energy $0.26\,eV$ and $0.80\,eV$. From the electric field distributions shown in insets, we also find the two deep transmission dips at $11.66\,THz$ and $20.51\,THz$ correspond to the same first-order graphene plasmon resonance, namely the dipole resonance, which is a bright mode. In addition, when the graphene ribbon possesses the Fermi energy $0.26\,eV$, there is another very slight transmission dip at about $20\,THz$, which is the second order mode and is a dark mode (see inset in Fig. 2(a)). These imply that graphene ribbon respectively electrically doped by Fermi energy $0.26\,eV$ and $0.80\,eV$ can support a bright mode and a dark mode operating at about $20\,THz$. So, one can expect EIT can be induced in a combination of the two same ribbons doped by different Fermi energy $0.26\,eV$ and $0.80\,eV$.

Then, we investigated the situation of arrays of symmetric graphene ribbon pairs with two ribbons doped by different Fermi energy $0.26\,eV$ and $0.80\,eV$ with $p=1360\,nm$, ${\varepsilon }_{1}=1$, $a=580\,nm$, and $d=10\,nm$. As one expect, an EIT spectral response with a transparency window located at a central frequency of $20.51\,THz$ (peak C) between two transmission dips at $19.56\,THz$ (dip B) and $21.68\,THz$ (dip D) is observed from Fig. 2(c). In addition, one can see another transmission dip at $9.42\,THz$ (dip A). In order to confirm the mechanism behind the electromagnetic induced transparency of this structure, the distributions of the $\hat{{\rm{z}}}$ component of electric field on the cutting plane perpendicular to $\hat{{\rm{z}}}$ direction at a distance of $10\,nm$ beneath graphene sheet in a unit cell at the transmission dips A, B, D, and peak C are shown in Fig. 2(d), where the graphene ribbon with lower Fermi energy $0.26\,eV$ is in the right (the dark ribbon) and another ribbon with higher Fermi energy $0.80\,eV$ is in the left (the bright ribbon). We compare the electric field distribution at about $20\,THz$ for arrays of single graphene ribbon (Fig. 2(a,b)) and arrays of graphene ribbon pairs (Fig. 2(d)). As for the arrays of single graphene ribbon, the bright mode at $20\,THz$ is strongly excited by the incident wave with a high electric field forming at its end facets (Fig. 2(b)). On the contrary, the dark mode at $20\,THz$ is weakly excited by the incident wave with a very weak electric field distribution (Fig. 2(a)). However, by placing two graphene ribbons together with $10\,nm$ apart, the electromagnetic field of the dark mode in the dark ribbon is greatly enhanced but the electromagnetic field in the bright ribbon is suppressed with a much weaker electric field at its ends (C in Fig. 2(d)). The enhanced dark mode comes from direct near-field coupling between the bright and dark ribbons. The suppressed bright mode is attributed to the destructive interference between the two pathways: direct dipole excitation of the bright ribbon from the incident wave and excitation of the dark ribbon (by the bright ribbon) coupling back to the bright ribbon. These results visually confirm the EIT-like destructive interference and are consistent with the previous theoretical predictions.

**Figure 3:**
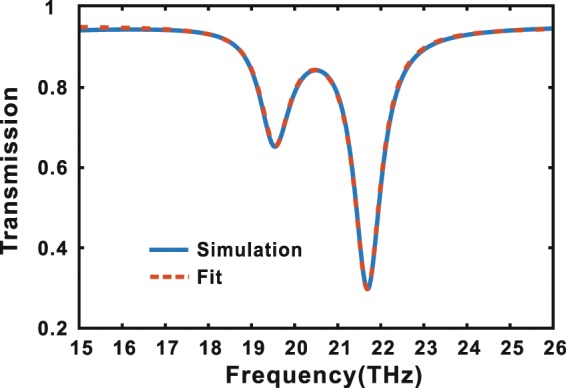
Simulation (blue solid line) and fitting (red dotted line) transmission of arrays of symmetric graphene ribbon pairs with two ribbons doped by different Fermi energy $0.26\,eV$ and $0.80\,eV$.

The EIT can be regarded as an analogue of Fano-type resonance. To further confirm the EIT in the structure, we fit our simulation results with the general form of the Fano-type resonance, which can be written as^58–60^

7 $$T\propto \sum _{i}\,\frac{{({\varepsilon }_{i}+{q}_{i})}^{2}}{1+{\varepsilon }_{i}^{2}},({\varepsilon }_{i}=\frac{\omega -{\omega }_{i}}{{\gamma }_{i}/2},i=1,2),$$

where ${\omega }_{i}$, ${\gamma }_{i}$ and ${q}_{i}$ ($i=1,2$) denote the resonance frequency, radiation damping and Breit-Wigner-Fano coupling coefficient for indicating the feature of the bright and dark modes in the bright and dark ribbons, respectively. Table 1 lists the specific values of the fitting parameters. The simulation and fitting results are shown in Fig. 3, where the blue solid line indicates the simulation result, and the red dotted line indicates the fitting result. From Fig. 3, one can see that the simulation and fitting results are consistent, which further confirm the EIT of this structure.

**Table 1:** Fitting parameter table.

Parameter	value	Parameter	value	Parameter	value
${\omega }_{1}$/$2\pi (THz)$	19.53	${\gamma }_{1}$/$2\pi (THz)$	0.825	*q*_1_	−0.038
${\omega }_{2}$/$2\pi (THz)$	21.69	${\gamma }_{2}$/$2\pi (THz)$	0.755	*q*_2_	0.001

In order to investigate the characteristics of the EIT of this structure, we sweep a few parameters, including the Fermi energy ${E}_{F1}$ and ${E}_{F2}$ of the bright and dark ribbons, ribbon width $a$, the dielectric constant ${\varepsilon }_{1}$ of surrounding medium, the periodic interval $p$ and the ribbon edge to edge distance $d$, respectively. It is necessary to stress that in each group of simulations, all the other parameters and conditions are kept the same as the above simulations.

**Figure 4:**
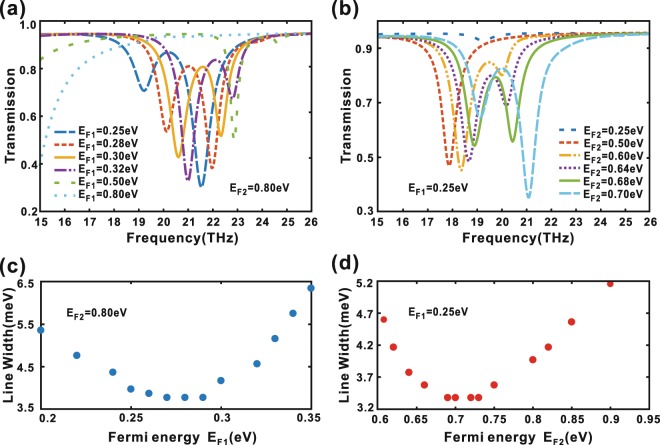
(**a**) Transmission spectra of arrays of symmetric graphene ribbon pairs with the ${E}_{F1}$ varying from $0.25\,eV$ to $0.80\,eV$, and ${E}_{F2}=0.80\,eV$. (**b**) Transmission spectra of arrays of symmetric graphene ribbon pairs with the ${E}_{F2}$ varying from $0.25\,eV$ to $0.70\,eV$, and ${E}_{F1}=0.25\,eV$. (**c**) The Line Width of the transparency window as a function of the varied ${E}_{F1}$ while ${E}_{F2}=0.80\,eV$. (**d**) The Line Width of the transparency window as a function of the varied ${E}_{F2}$ while ${E}_{F1}=0.25\,eV$.

We first calculated the transmission spectra as functions of frequency when the Fermi energy ${E}_{F1}$ of the dark ribbon varies from $0.25\,eV$ to $0.80\,eV$ with $p=1360\,nm$, $a=580\,nm$, $d=10\,nm$, and ${E}_{F2}=0.80\,eV$, as shown in Fig. 4(a), respectively. From Fig. 4(a), it can be observed that the EIT is very sensitive to the Fermi energy ${E}_{F1}$ of the dark ribbon when ${E}_{F2}$ is fixed. One key parameter of the EIT is full line width of the transparency window, which is defined as the energy difference between the antiresonance dip and adjacent peak^24^. As shown in Fig. 4(c), with the increase of Fermi energy ${E}_{F1}$, the line width of the transparency window exhibits the trend from widen to narrow, and then to widen again until the EIT effect disappears, which indicates there is an optimized ${E}_{F1}$ for obtain EIT. This can be understood through the following qualitative analysis. ${E}_{F1}$ has a specific solution ${E}_{F10}$ satisfying Eq. (5) for a fixed ${E}_{F2}=0.80\,eV$. When ${E}_{F1}$ is equal to ${E}_{F10}$, the near field coupling and interference between the two bright and dark modes are the strongest, so the line width of the transparency window is the narrowest. As the deviation of ${E}_{F1}$ departing form ${E}_{F10}$ gets bigger and bigger, the resonate frequencies of the dark mode deviates more from that of the bright mode and the corresponding coupling and interference between the two bright and dark modes become weaker and even disappear, so the line width of the transparency window becomes wider. The phenomenon when the Fermi energy ${E}_{F2}$ of the bright ribbon varies from $0.25\,eV$ to $0.70\,eV$ with $p=1360\,nm$, $a=580\,nm$, $d=10\,nm$, and ${E}_{F1}=0.25\,eV$ is similar and can be explained similarly, as shown in Fig. 4(b,d), respectively.

**Figure 5:**
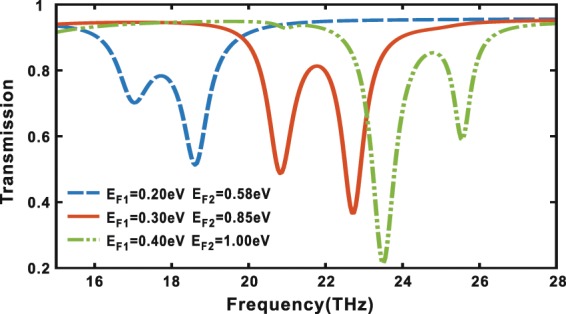
Transmission spectra of arrays of symmetric graphene ribbon pairs with the ${E}_{F1}$ varying from $0.20\,eV$ to $0.40\,eV$ and ${E}_{F2}$ correspondingly varying from $0.58\,eV$ to $1.00\,eV$.

From the results above and Eq. (5), one can predict that the transparency window of the EIT can be dynamically tunable when the Fermi energy of ${E}_{F1}$ of the dark ribbon and ${E}_{F2}$ of the bright ribbon are simultaneously and correspondingly changed. Figure 5 shows the transmission spectra of arrays of symmetric graphene ribbon pairs with the ${E}_{F1}$ varying from $0.20\,eV$ to $0.40\,eV$ and ${E}_{F2}$ correspondingly varying from $0.58\,eV$ to $1.00\,eV$. It can be seen from Fig. 5 that frequency of the transparency window has a remarkable blue shift from $17.74\,THz$ to $24.80\,THz$ as the ${E}_{F1}$ increases from $0.20\,eV$ to $0.40\,eV$ and ${E}_{F2}$ correspondingly varying from $0.58\,eV$ to $1.00\,eV$.

**Figure 6:**
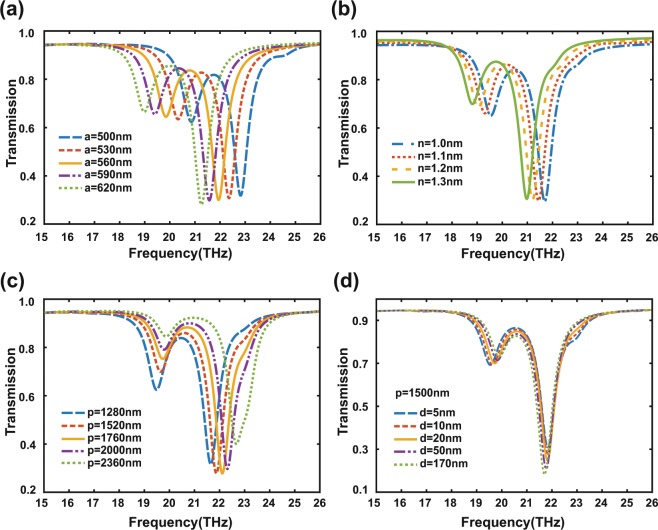
Transmission spectra of arrays of symmetric graphene ribbon pairs for different (**a**) $a$, (**b**) $n$ and (**c**) $p$, respectively. (**d**) Transmission spectra of graphene ribbon pairs for only different $d$ while $p=1500\,nm$.

Figure 6(a) shows the calculated transmission as a function of frequency for different $a$ with $p=1360\,nm$, $d=10\,nm$, ${E}_{F1}=0.26\,eV$, and ${E}_{F2}=0.80\,eV$. One can see that the transparency window band of EIT is very sensitive to $a$. The transparency window occurs a red shift as $a$ increases, which is consistent with Eq. (5). The surrounding medium with dielectric constant ${\varepsilon }_{1}$ also has influence on the frequency of transparency window. From Eq. (5), one can find that a lager ${\varepsilon }_{1}$ leads to a smaller resonant frequency of the plasmon resonance mode. So, a red shift of transparency window with increased $n$ ($n=\sqrt{{\varepsilon }_{1}}$) can be see, as demonstrated in Fig. 6(b). The transmission as a function of frequency for different $p$ with $a=580\,nm$, $d=10\,nm$, ${\varepsilon }_{1}=1$, ${E}_{F1}=0.26\,eV$, and ${E}_{F2}=0.80\,eV$ is calculated and shown in Fig. 6(c). Figure 6(c) indicates that the frequency of transparency window has a blue shift with increased $p$. This can be understood through the following qualitative analysis. The duty ratio of structure decrease as the $p$ increases. According to the equivalent medium theory^61^, a smaller duty ratio of structure leads to a smaller equivalent refractive index, and result in a larger resonance frequency of the dark mode. In order to discuss the influence of $d$ on EIT, we increase the periodic interval $p$ to reduce the interaction between adjacent periodicity and calculate the transmission spectra with $p=1500\,nm$, $a=580\,nm$, ${\varepsilon }_{1}=1$, ${E}_{F1}=0.26\,eV$, and ${E}_{F2}=0.80\,eV$. As shown in Fig. 6(d), one can see that the transparency window remains almost unchanged when the $d$ is increased from $5\,nm$ to $170\,nm$. This is because the $d$ in this range is much smaller than the wavelength of the bright mode or dark mode, and near field coupling and interference between the bright mode and dark mode can still occur.

**Figure 7:**
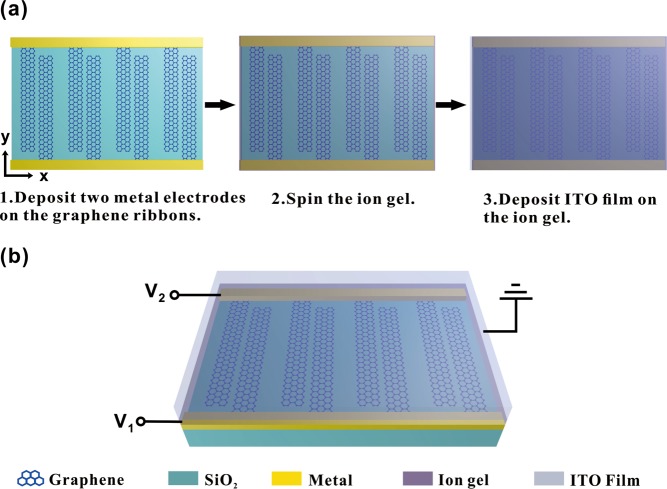
(**a**) Schematics showing the process for realizing Fermi energy of graphene ribbons physically controlled by two electrostatic top gatings with an ion gel gating scheme. (**b**) Schematic diagram of practical device.

Finally, we discuss how the Fermi energy ${E}_{F1}$ and ${E}_{F2}$ can be physically controlled in the graphene-based tunable EIT devices. As for practical applications, the Fermi energy ${E}_{F1}$ and ${E}_{F2}$ can be physically controlled by two electrostatic top gatings (changing the external gate voltage) with an ion gel gating scheme^62,63^. The detailed process for fabrication is shown in Fig. 7(a). First, a graphene film is etched by electron beam into arrays of graphene ribbon pairs staggered along the $\hat{{\rm{y}}}$ direction and form a structure similar to the interdigital electrodes^28^. Then, two metal electrodes are deposited and respectively contact on one ribbon of graphene ribbon pairs along the $\hat{{\rm{x}}}$ direction. Next, the ion gel (the mixture of P(VDF-HFP) and [EMIM][TFSI]) is spun on arrays of graphene ribbon pairs. After that, a transparency Indium Tin Oxides (ITO) film is deposited on the ion gel as a top gate electrode. The schematic diagram of the practical device is shown in Fig. 7(b). By changing two external gate voltages ${V}_{1}$ and ${V}_{2}$, the Fermi energy ${E}_{F1}$ and ${E}_{F2}$ of two graphene ribbons in a ribbon pair can be physically controlled, respectively.

## Conclusions

In conclusion, we demonstrate that EIT can be induced by arrays of graphene ribbon pairs without structurally or spatially asymmetry. By changing two external gate voltages, the Fermi energy of two graphene ribbons in a ribbon pair can be physically controlled, respectively, so the EIT can be tuned without refabricating the physic structure. At the same time, in contrary to EIT in a structurally or spatially asymmetry structure where structurally asymmetric is introduced by deliberately breaking the element symmetry in shape as well as in size, EIT in the symmetry structure facilitates the design and fabrication of the structure. In addition, since the EIT is a Fano-type response, the work regarding to EIT in the structurally symmetric could provide a fresh contribution to a more comprehensive physical understanding of Fano resonance.
